# Experimental Assessment of Human–Structure Interaction in an Urban Pedestrian Footbridge with Multiaxial Dynamic Sensitivity

**DOI:** 10.3390/s26154780

**Published:** 2026-07-28

**Authors:** Bryan Castillo, Angie L. Arango, Johannio Marulanda, Colin Caprani, Peter Thomson

**Affiliations:** 1 Faculty of Engineering, Universidad Santiago de Cali, Santiago de Cali 760001, Colombia; 2 School of Civil and Geomatic Engineering, Universidad del Valle, Santiago de Cali 760001, Colombia; angie.lorena.arango@correounivalle.edu.co (A.L.A.); johannio.marulanda@correounivalle.edu.co (J.M.); peter.thomson@correounivalle.edu.co (P.T.); 3 Department of Civil and Environmental Engineering, Monash University, Melbourne, VIC 3800, Australia; colin.caprani@monash.edu

**Keywords:** human–structure interaction, pedestrian footbridges, human-induced vibrations, pedestrian density, multiaxial dynamic sensitivity

## Abstract

**Highlights:**

**What are the main findings?**

- Human–structure interaction (HSI) effects are primarily concentrated around the lateral fundamental frequency of the footbridge.
- Structural damping increases exponentially with pedestrian load density, significantly influencing the dynamic response.

**What are the implications of the main findings?**

- Serviceability assessments of footbridges should explicitly account for multiaxial vibration limits, especially under moderated pedestrian densities.
- Full-scale experimental data can enhance the reliability of vibration-based design methods for low-frequency pedestrian bridges.

**Abstract:**

Recent advances in structural engineering have enabled lightweight and slender footbridges. However, these structures are often susceptible to excessive vibrations induced by pedestrian activities, primarily impacting urban footbridge serviceability. Most studies evaluate human–structure interaction (HSI) uniaxially based on peak dynamic sensitivity. Nevertheless, this approach may overlook multidirectional mechanisms in footbridges with similar vertical and lateral modal properties. This study presents an extensive experimental assessment of HSI effects on a functional urban pedestrian bridge, known as the Premier-Footbridge, which exhibits clear multiaxial dynamic sensitivity within the frequency range associated with human walking. The structure was characterized by using operational modal analysis (OMA) based on ambient vibration measurements. Subsequently, a comprehensive human gait campaign evaluated different pedestrian density loads (PDL) and gait conditions, including synchronized, non-synchronized, and random walking. The results show that the multiaxial interaction effects were predominantly concentrated around the first lateral vibration mode (1.07 Hz), despite concurrent dynamic sensitivity in both lateral (1.07 Hz) and vertical (1.98 Hz) directions. Increasing PDL levels led to higher vibration amplitudes and measurable changes in HSI-related structural dynamics, including apparent damping and pedestrian step frequency. The experimental results indicated an apparent increase in effective structural damping with increasing pedestrian density, as identified through nonlinear trend fitting of the measured modal response parameters. Despite the multiaxial dynamic sensitivity of the structure, lateral vibrations remained the dominant factor governing the serviceability response, even under moderate PDL conditions.

## 1. Introduction

The dynamic interaction between loads induced by human activities and structural responses is a key factor governing the serviceability performance of civil engineering structures. Excessive vibrations caused by pedestrian activities have been widely reported in several structural systems, including stairs [1,2], grandstands [3,4], floors [5,6], and pedestrian bridges [7,8,9,10,11,12]. Among these structures, pedestrian footbridges are particularly susceptible to vibration serviceability problems due to their typically slender configurations, long spans, and relatively low structural mass and damping. Recent architectural and engineering trends have promoted the use of lightweight materials and optimized structural designs, which often result in more flexible structures. Although these strategies improve structural efficiency, they also increase the dynamic sensitivity of footbridges to pedestrian-induced loads [13,14]. While impulsive human actions may generate high-intensity loads [15,16], pedestrian walking remains the most frequent source of dynamic excitation. This is mainly due to the proximity between the frequency content of human gait, typically ranging between 1.60 and 2.40 Hz in the vertical direction and 0.70–1.30 Hz in the lateral direction, and the fundamental frequencies commonly found in pedestrian bridges [2,17].

When the vibration frequency of a footbridge approaches the frequency content of pedestrian walking, interaction mechanisms may develop between pedestrians and the structure. In such conditions, pedestrians may unconsciously adapt their walking rhythm to structural vibrations, which can promote synchronization effects and lead to an amplification of the structural response. These interaction mechanisms can significantly affect vibration serviceability and pedestrian comfort [13,18,19]. Human–structure interaction (HSI) phenomena arise from the complex coupling between pedestrian motion and structural vibrations. These interactions involve several mechanisms, including structure-to-human interaction (S2HI), where structural vibrations influence pedestrian gait dynamics [20,21]; human-to-structure interaction (H2SI), associated with changes in structural dynamic properties caused by the presence of pedestrians [22,23]; and human-to-human interaction (HHI), which describes interactions among pedestrians walking in groups [22,23,24]. Recent studies have also identified internal human–human interaction (I-HHI) effects, in which structural vibrations may indirectly influence synchronization and interaction mechanisms between pedestrians. Together, these mechanisms define the overall HSI effects governing the vibrational behavior of pedestrian bridges [25,26].

Early studies on pedestrian-induced vibrations often considered walking loads acting on rigid surfaces, neglecting the interaction between pedestrians and structural motion [27]. More recent research has explored HSI through controlled laboratory experiments using instrumented treadmills capable of reproducing structural motion in one or more directions [25,28,29,30,31]. Although these approaches provide important insight into gait dynamics, laboratory conditions may influence pedestrian behavior, limiting the representativeness of the results compared with real operational environments [30]. Several large-scale experimental investigations have therefore been conducted on existing pedestrian bridges under realistic pedestrian loading conditions. A well-known example is the Millennium Bridge, where excessive lateral vibrations were observed during its opening due to pedestrian synchronization effects, as documented by Dallard et al. [32] and later studies [33,34]. Similar vibration phenomena have been reported in other pedestrian bridges, including the Solferino Bridge [35] and the Changi Airport Bridge [36,37]. Additional studies have also examined pedestrian-induced vibrations in structures such as the Wuhan Yangtze River Bridge [38] and the Ponte del Mare [39], among others [12,40,41]. Among recent experimental investigations, Van Nimmen et al. [42,43] conducted one of the most comprehensive measurement campaigns on the Eeklo Footbridge, providing a notable benchmark dataset for the study of HSI effects.

In recent decades, vibration monitoring based on ambient excitation has become an important tool for identifying the operational dynamic characteristics of civil engineering structures, particularly in lightweight and dynamically sensitive systems [44,45]. Output-only operational modal analysis (OMA) techniques allow the identification of modal properties under real operating conditions without interrupting structural service, providing valuable information for structural health monitoring, serviceability assessment, and model calibration [46]. Previous studies have demonstrated the effectiveness of ambient and forced vibration measurements for evaluating the dynamic behavior of structures subjected to operational loads and environmental disturbances [47]. In particular, applications in vibration-based monitoring have highlighted the relevance of experimental dynamic characterization for understanding structural performance under realistic excitation scenarios. However, despite these advances, relatively limited attention has been given to the experimental assessment of multiaxial HSI effects in pedestrian footbridges operating under realistic pedestrian density load (PDL) conditions. Most existing investigations have primarily focused on the dominant vibration direction [48,49], potentially overlooking coupled dynamic mechanisms associated with structures exhibiting similar lateral and vertical modal sensitivities, such as the multiaxial behavior investigated in the present research.

**Figure 1:**
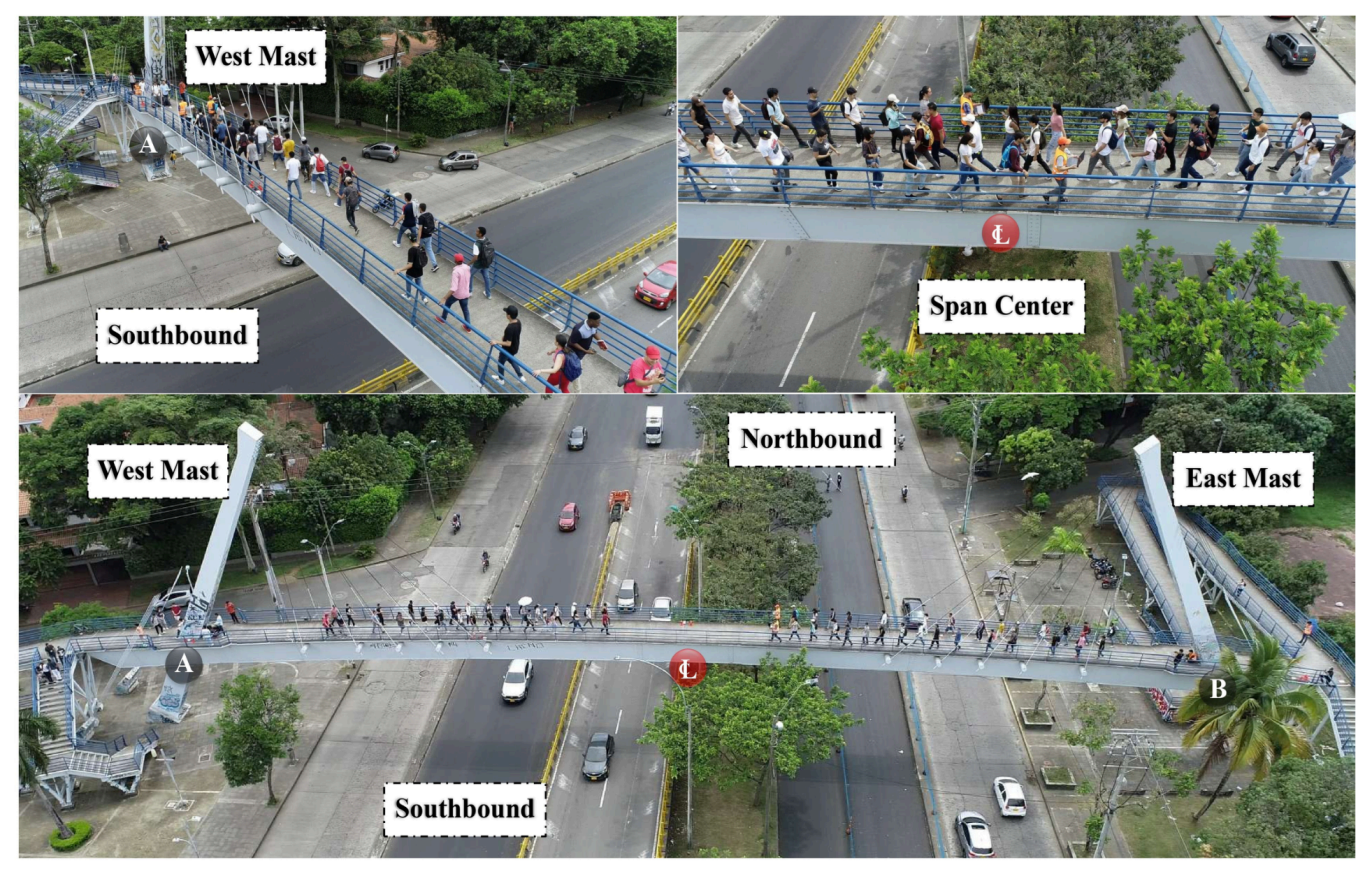
Large-scale gait test on the Premier Footbridge. View of west mast (**top-left**), pedestrians crossing on the footbridge span center (**top-right**), and aerial view of the Premier footbridge from south to north (**bottom**).

Therefore, this study presents a comprehensive experimental assessment of HSI effects in a functional urban pedestrian bridge with multiaxial dynamic sensitivity. The investigated structure, known as the Premier Footbridge, is located in Santiago de Cali, Colombia, and exhibits fundamental frequencies of 1.07 Hz in the lateral direction and 1.98 Hz in the vertical direction, both within the characteristic frequency range of human walking. The footbridge was first characterized by using OMA based on ambient vibration measurements. Subsequently, an extensive human gait experimental campaign was conducted under different PDL and gait conditions, including synchronized, non-synchronized, and random walking. The views of this large-scale measurement campaign are shown in Figure 1. Beyond the experimental characterization of the structure, this assessment contributes to the understanding of pedestrian–structure interaction mechanisms by experimentally assessing both active and passive HSI effects. In addition, the influence of PDL levels on the dynamic properties and vibration response of the footbridge is quantified, providing new experimental evidence on how crowd loading conditions modify modal characteristics and serviceability-related vibration levels in dynamically sensitive footbridges.

The resulting Premier Footbridge dataset provides useful experimental evidence for understanding pedestrian–structure interaction mechanisms and evaluating vibration serviceability in dynamically sensitive footbridges. The remainder of this paper is organized as follows. Section 2.1 describes the structural characteristics and dynamic identification of the Premier Footbridge. Section 2.2 presents the measurement system, experimental setup, and human gait testing protocol. Section 3 discusses the results obtained from the experimental campaign, and Section 4 presents the main conclusions of the study.

## 2. Materials and Methods

### 2.1. Description of the Structure: Premier Footbridge

**Figure 2:**
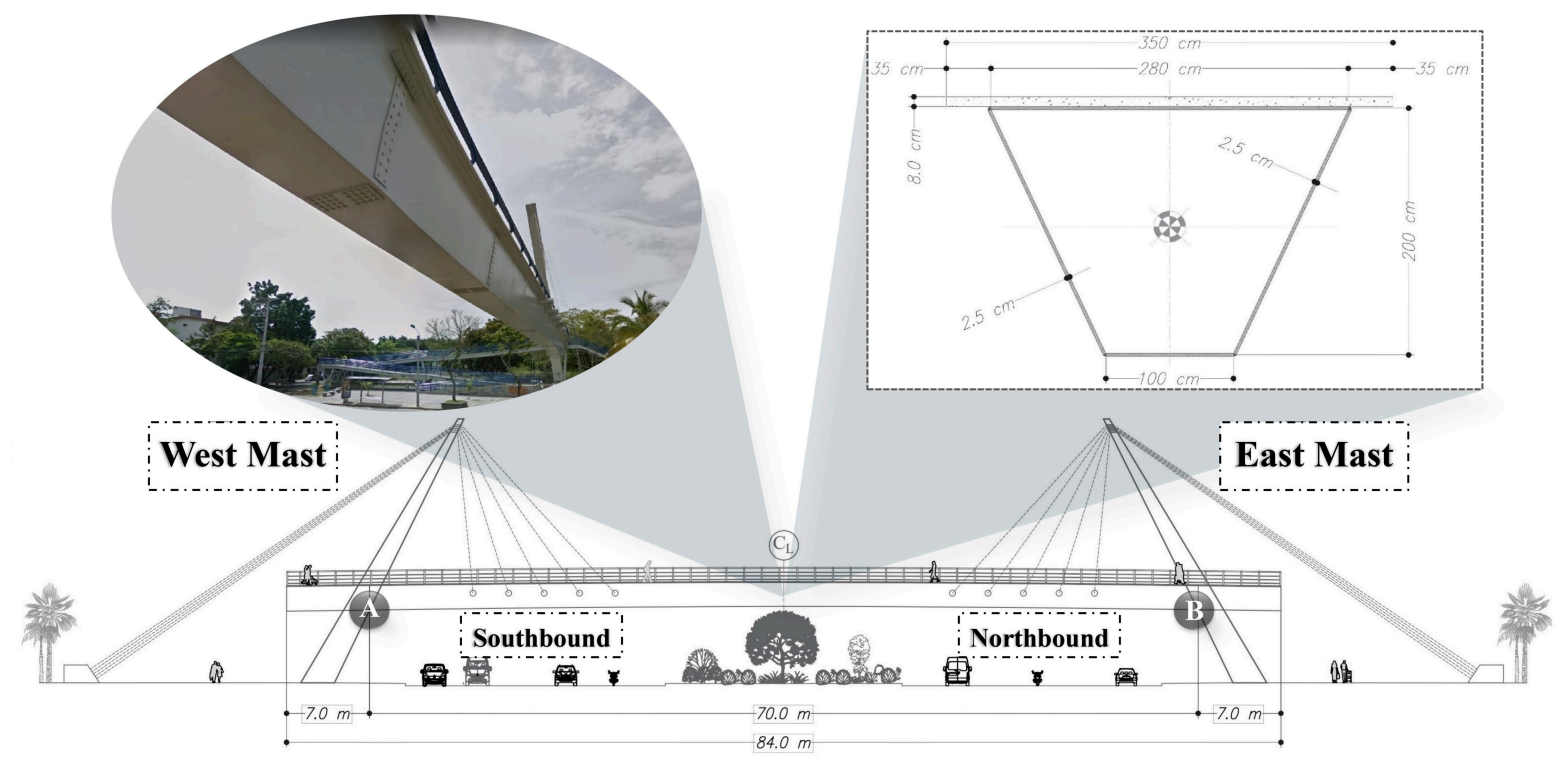
Premier footbridge lower view and cross section (**top**); and schematic side view (**bottom**).

The Premier Footbridge has a total length of 84.0 m, consisting of a central main span of 70.0 m and two lateral cantilever spans of 7.0 m each, as shown in Figure 2 (bottom). The cross section of the footbridge consists of an assembled steel box girder with a height of 2.0 m, spaced 3.50 m apart and with a plate thickness of 2.50 cm, supporting a concrete slab with a thickness of 80.0 mm (Figure 2, top). The structure is supported by two mains inclined masts of 64.0°, which distribute ten tensioning strands with a diameter of 2.54 cm (five on each side near the respective mast), anchored to two external concrete blocks. The total mass of the footbridge is approximately 614.0 tons, composed of 32.0 tons of steel elements and 582.0 tons of concrete elements. An OMA was conducted to obtain experimental dynamic information for the development of a detailed finite element (FE) model, based on the as-built plans and field data, as detailed in Section Operational Modal Analysis (OMA) and Modelling of the Structure.

#### Operational Modal Analysis (OMA) and Modelling of the Structure

**Figure 3:**
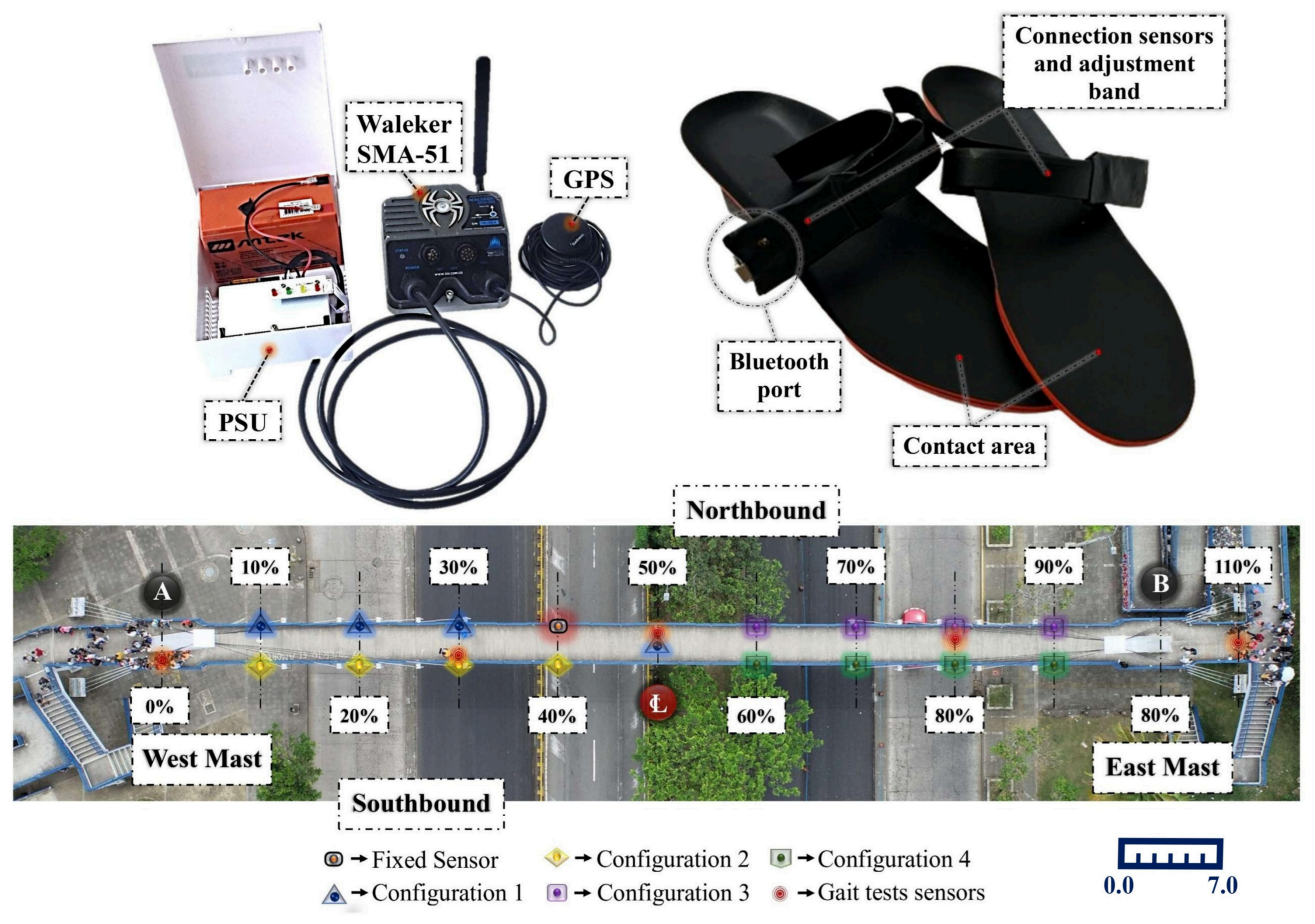
Measurement Equipment and sensors for test subjects (TSs), Waleker SMA-551 triaxial acceleration sensors (**Top-left**); insole pressure sensor Stridalyzer Insight by ReTiSense (**Top-Right**); and sensors location during operational modal analysis and gait test, top view (**Bottom**).

**Figure 4:**
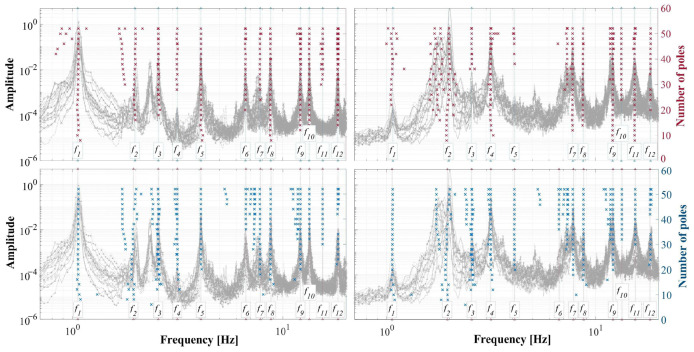
Operational Modal Analysis on Premier footbridge. Stabilization diagram of the OMA using NExT-ERA compared with PP location, vertical and lateral direction (**Top**, red). Stabilization diagram of the OMA using SSI compared with PP location, vertical and lateral direction (**Bottom**, blue).

An extensive experimental measurement campaign was conducted to determinate the operational modal properties of Premier Footbridge. The identification of the system was performed using output-only system identification techniques based on ambient vibrations, mainly generated by service traffic and environmental disturbances. The structural accelerations were processed using two OMA algorithms: reference-based data-driven stochastic subspace identification (SSI) [50], and natural excitation technique combine with an eigensystem realization algorithm (NExT-ERA) [51]. Ambient vibrations of the Premier Footbridge were measured along the entire length of the structure using Waleker SMA-51 Sensors, placed at 7.0 m intervals, as shown in Figure 3 (Bottom), with the position configuration shown in Figure 3 (Top-left). A total of twenty-one measurement points were instrumented in the vertical and lateral directions, using five experimental configurations with a sampling rate of 125.0 Hz, while maintaining one reference sensor in all configurations to ensure data correlation. Using experimental structural response data, twelve vibration modes with natural frequencies up to 15.0 Hz were identified using the OMA algorithms. A sensitivity analysis of stabilization of the system poles used in the NExT–ERA and SSI methods was performed and compared with the frequency-domain peak-picking (PP) method, as shown in Figure 4. Natural frequencies ${\hat{f}}_{j}$ and ${\overset{˘}{f}}_{j}$, and damping ratios $\hat{\xi }$ and $\overset{˘}{\xi }$ were identified using NExT-ERA and SSI algorithms, respectively, are presented in Table 1. All algorithms were configurated with a time window length of $2.0^{12}$ (Hamming type), an overlap of 50.0%, and a frequency resolution of 0.01 Hz (combine between instrument resolution devices and preprocessing of the algorithms).

**Figure 5:**
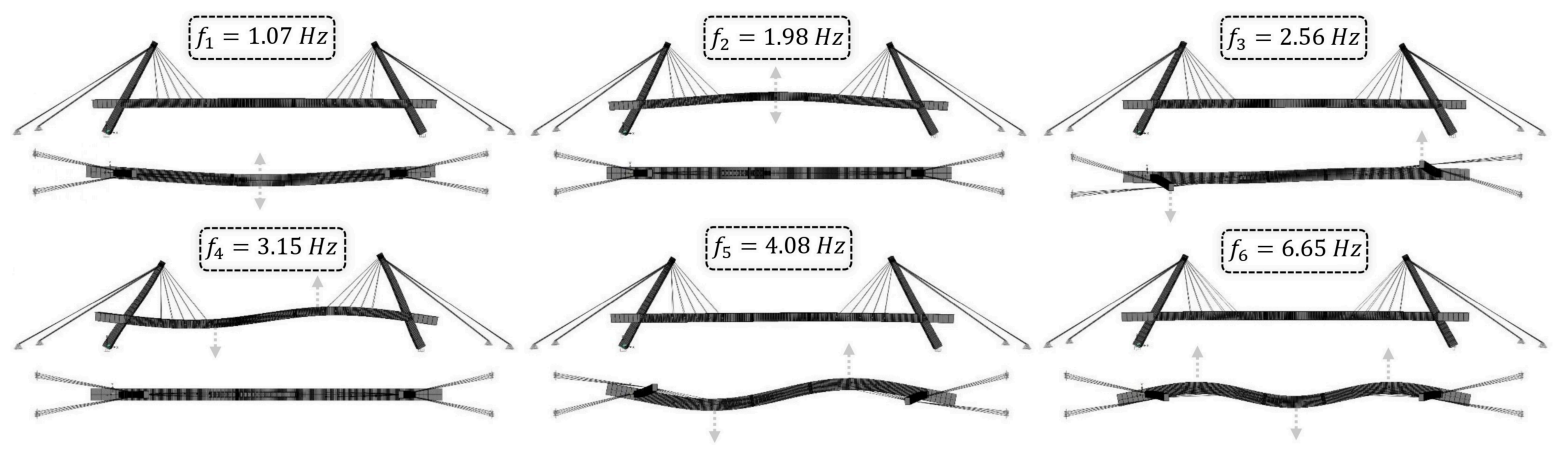
Top and lateral views of the first six natural modes of the Premier footbridge: Mode 1 → $f_{1}$ = 1.07 Hz; Mode 2 → f_2_ = 1.98 Hz; Mode 3 → f_3_ = 2.56 Hz; Mode 4 → f_4_ = 3.15 Hz; Mode 5 → f_5_ = 4.08 Hz; and Mode 6 → f_6_ = 6.65 Hz.

A detailed FE model of the Premier Footbridge was developed based on the as-built plans and field information obtained during technical inspections. The analytical model was calibrated using the experimentally identified modal properties. The calibration parameters included the elastic modulus of the steel, the moments of inertia of the main girder and masts, the effective area of the steel tension strands, and the thickness of the deck slab. Table 1 shows the outstanding agreement between the experimentally identified modal characteristics and those predicted by the calibrated FE model. The relative errors in the natural frequencies of the first seven vibration modes are limited to 1.59 while the Modal Assurance Criterion (MAC) values, calculated using Equation (1), are all close to 1.0. In addition, the parameters ℣ and Ƚ exhibited values greater than 1000 in the predominant vibration direction, indicating a strong directional tendency in the structural dynamic response and confirming the dominance of HSI effects along that vibration mode. The first six mode shapes are presented in Figure 5, illustrating the alternation between lateral and vertical vibration modes.

(1) $${MAC}_{a-e}=\frac{{\left(\Phi_{a}^{T}\Phi_{e}\right)}^{2}}{\Phi_{a}^{T}\Phi_{a}\Phi_{e}^{T}\Phi_{e}}$$

(2) $$m_{j,e}={max(\left|\Phi_{j,e}\right|)}^{-2}$$

In order to interpret the structural response, Table 1 lists the apparent modal masses in the vertical ($m_{\Phi ,V}$) and lateral ($m_{\Phi ,L}$) directions, which were calculated using Equation (2). Where $\Phi_{j,e}$ denotes the components in direction e (lateral l or vertical v) of the mass-normalized mode shape $\Phi_{j}$. For a purely vertical or lateral mode, $m_{\Phi ,V}$ or $m_{\Phi ,L}$ corresponds to the proportion of physical mass of the structure employed in that mode [52]. Consequently, the smaller $m_{\Phi -j,e}$, the more sensitive mode Φ-j is to dynamic excitation in direction e. The values for $m_{\Phi ,V}$ and $m_{\Phi ,L}$ reported in Table 1 indicate that modes 1, 5 and 6 are purely lateral bending modes (Ƚ), while modes 2, 4 and 7 are purely vertical bending modes (℣); associated with a low apparent lateral ($m_{\Phi ,L}$) and vertical ($m_{\Phi ,V}$) modal mass, respectively. Moreover, mode 3 is associated with the dynamic behavior of the supporting masts, exhibiting a stronger lateral response.

Although the modal parameters identified using SSI and NExT-ERA showed strong agreement, small differences between both methods were observed due to variations in the identification procedures, noise sensitivity, and operational excitation treatment. Nevertheless, these discrepancies remained limited, confirming the consistency of the estimated modal parameters.

### 2.2. Experimental Test

A large-scale experimental campaign involving synchronized and non-synchronized gait tests was conducted under different pedestrian-induced loading conditions. This section describes the assessment procedure, including the measurement equipment and experimental setup, data processing methodology, characteristics of test subjects (TSs), and the configuration of the experimental tests.

#### 2.2.1. Measurement Equipment and Setup

Premier footbridge accelerations were measured using wireless triaxial acceleration sensors (Waleker SMA-551 (SSI, Colombia), range ± 2.0 g, least significant bit (LSB) of 3.815 × 10^−6^ g, [53]) with a sample time of 125.0 Hz. The sensor configuration, including its global positioning system (GPS) and power supply unit (PSU), is shown in Figure 3 (Top-left). A maximum of five sensors were used under five experimental configurations, maintaining a fixed sensor in all configurations to correlate the data implemented in the OMA algorithms. In addition, a sixth experimental configuration was implemented for the pedestrian gait-induced excitation tests, as shown in Figure 3 (Bottom) with red marker-points.

For each human gait-induced load test described in Section 2.2.4, two TSs were instrumented with a pair of insole pressure sensors (Stridalyzer Insight developed by ReTiSense) [54]. These devices, shown in Figure 3 (Top-Right), operate with an adjustable sampling frequency between 1.0 and 50.0 Hz. Each sensor integrates a three-dimensional inertial measurement unit (IMU), a gyroscopic sensor, and ten micro-load sensors distributed across the contact surface of the device.

#### 2.2.2. Data Processing

Structural acceleration signals were preprocessed in MATLAB (v.2024a) as follows: (1) the offset was removed using detrend function; (2) the sampling frequency remained at 125.0 Hz and a low-pass filter with a cut-off frequency of 120.0 Hz was applied; and (3) A high-pass filter was applied using a fourth-order Butterworth filter with a cut-off frequency of 0.10 Hz. Based on this preprocessing, an operational modal analysis (OMA) was conducted using two algorithms (SSI and NExT-ERA) to identify the dynamic properties of the Premier footbridge, as described in Section 2.1. The processed signals were also used to evaluate the dynamic effects of human-induced gait loads on the structure, as discussed in Section 3.1.

Human-induced gait loads acquired with insole pressure sensor Stridalyzer Insight were preprocessed in Microsoft Office (MOf) and MATLAB as follows: (1) The human-induced gait load data were downloaded from online database ReTiSense platform with a sampling frequency of 50.0 Hz in MOf format; (2) the data were imported into MATLAB using the xlsread function and reorganized according to each event evaluated; (3) the sampling frequency remained at 50.0 Hz and a low-pass filter with a cut-off frequency of 30.0 Hz; and (4) A high-pass filter was applied using a fourth-order Butterworth filter with a cut-off frequency of 0.05 Hz. Based on this preprocessing, human-induced gait loads were analyzed to evaluate changes in the dynamic behavior of these loads under the influence of structural vibrations, as described in Section 3.2.

Synchronization between pedestrian-induced gait loads and the structural vibration of the Premier Footbridge during the loading events was achieved through a random impact generated by a TS wearing the insole pressure sensors near one of the triaxial accelerometers. This signal was simultaneously recorded by the sensor network through the GPS-based synchronization system.

#### 2.2.3. Test Subjects

The ID, height, body mass, and sex of the TSs are reported in Table 2. For the gait test involving a PDL between 0.005 and 0.357 $people\cdot m^{-2}$, the average height of the TSs was 1.73 m ± 6.40 cm, and the average body mass was 68.60 ± 13.80 kg. All pedestrians walked according to configuration of the tests described in Section 2.2.4 on the Premier Footbridge. In addition, the first twenty TSs, highlighted in gray in Table 2, also participated in the rigid-surface gait tests, as described in Section Rigid Surface Gait Test.

#### 2.2.4. Configurations of Gait Test

##### Rigid Surface Gait Test

**Figure 6:**
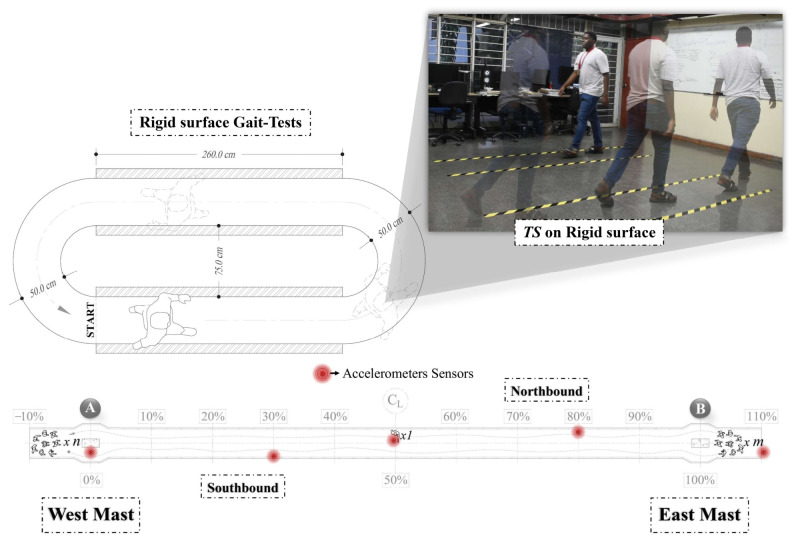
Configuration of rigid surface gait tests (**top**), and Experimental Gait-test on Premier Footbridge with triaxial accelerometers setup (**bottom**).

The rigid surface gait tests scenario consisted of a runway composed of two parallel lanes, each 2.60 m long and 0.50 m wide, with an inner spacing of 0.75 m between the lanes, as is shown in Figure 6 (Top). The runway was demarcated using yellow non-slip tape so that TSs could clearly identify the walking path. This configuration allows individuals to perform at least two consecutive linear steps. Additionally, the configuration ensured that TSs felt comfortable and were able to walk naturally.

Each TS performed two tests. In the first test, participants walked along the track for three complete laps (starting and finishing at the same point) with a target walking frequency close to 2.0 Hz. A metronome was used to control this condition, set at 120 BPM, which corresponds to a stepping frequency of approximately 2.0 Hz. The second test also consisted of three consecutive laps; however, in this case, each TS walked at their natural pace without metronome guidance. All TSs wore the Stridalyzer insole pressure sensors during both gait test scenarios.

##### Gait Tests on Premier Footbridge

The gait tests on the Premier Footbridge consisted of a complete pedestrian crossing starting and finishing at points A or B (turning around at the opposite end), as shown in Figure 6 (Bottom). A total of twenty-five tests were conducted on the footbridge. These gait tests were divided into synchronized tests, in which groups of pedestrians used metronomes to induce a gait frequency ($f_{g}$) close to 2.0 Hz, and non-synchronized tests in which pedestrians walked freely at their natural gait frequency. In addition, two random gait tests were conducted with pedestrian density loads (PDL) of 10.22% and 35.77% (0.102 and 0.358 $people\cdot m^{-2}$). All gait tests, including the walking condition, the initial number of TSs starting at points A or B, and the corresponding PDL values, as listed in Table 3. The Stridalyzer insole pressure sensors were worn by the same two pedestrians during the all-gait tests, except for tests 1 and 2, which involved a single pedestrian.

Furthermore, in tests 14, 19, and 24 one pedestrian remained in a passive state (standing) at the middle span of the structure (50.0%), denoted as (℄), while wearing the insole pressure sensors, as shown in Figure 6 (bottom).

## 3. Results and Discussion

### 3.1. Dynamic Performance of the Premier Footbridge

The dynamic performance of the Premier Footbridge was assessed using the structural accelerations recorded at 30.0% and 50.0% of the structural span. These locations were selected according to the sensor configuration described in Section 2.2.1 and the gait test configuration presented in Section Gait Tests on Premier Footbridge, which provided the most representative dynamic information of the structure, as described in Section Operational Modal Analysis (OMA) and Modelling of the Structure. All gait tests at each assessment point were analyzed in the frequency domain using the Cross-Power Spectral Density (CPSD) formulation presented in Equation (3), following the methodology proposed in [55,56].

(3) $${CPSD}_{xy}\left(\omega \right)=\sum_{m=-\infty }^{\infty }R_{xy}\left(m\right)e^{-j\omega m}\;;\;R_{xy}\left(m\right)=E\left\{x_{n+m}\cdot y_{n}^{*}\right\}\;\;\;\forall \;\left\{-\infty <n<\infty \right\}$$

**Figure 7:**
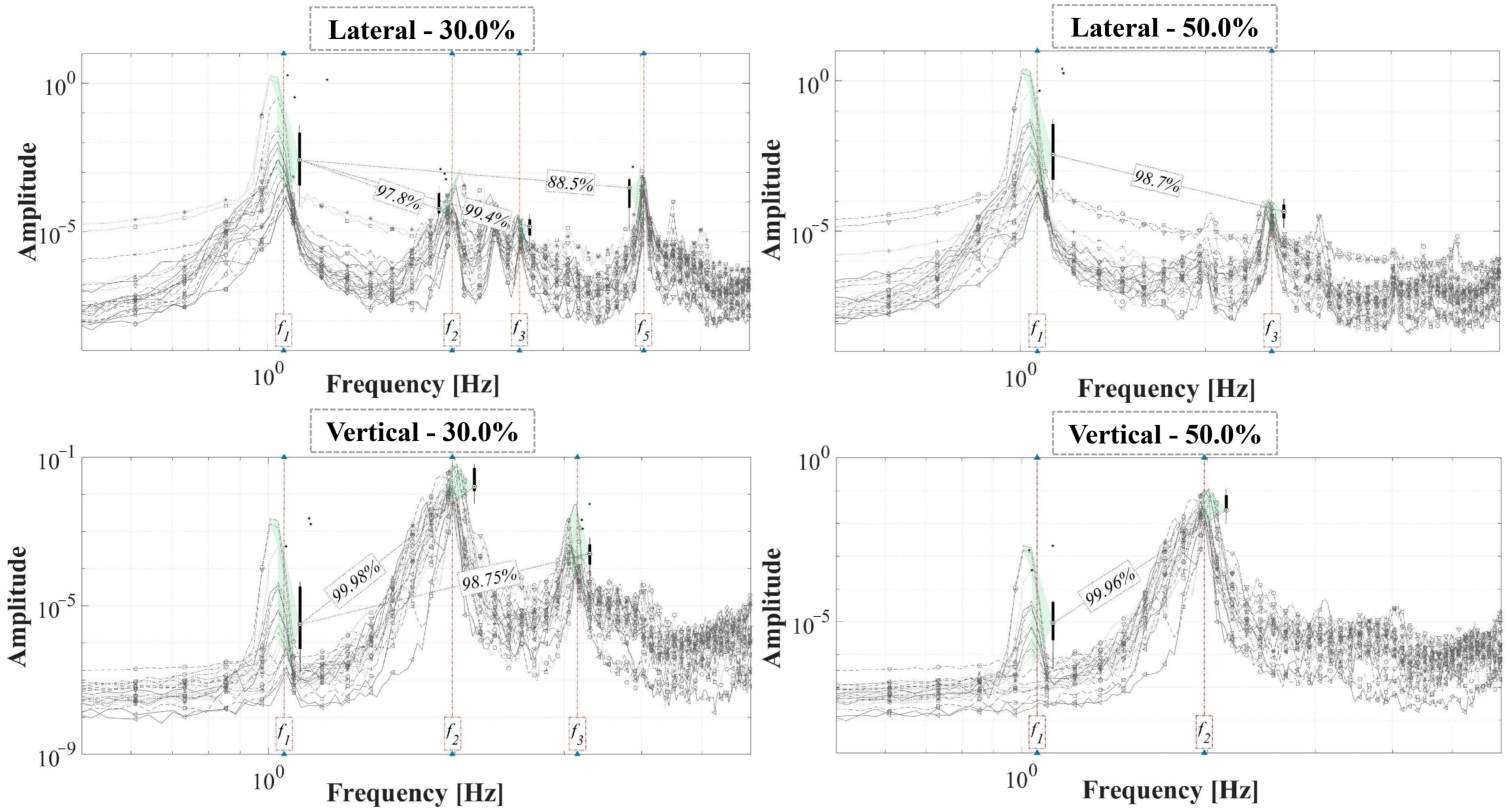
Dynamic response vertical and lateral of the Premier Footbridge under gait tests. Lateral dynamic response at 30.0% length (**Top-left**), lateral dynamic response at 50.0% length (**Top-right**), vertical dynamic response at 30.0% length (**Bottom-left**), and vertical dynamic response at 50.0% length (**Bottom-right**).

The CPSD frequency analysis of the gait tests for all evaluated PDL revealed that the lateral dynamic behavior at the 30.0% span location was predominantly governed by the fundamental frequency $f_{1}=1.07\;Hz$. The mean response amplitudes associated with this frequency were more than 88.50% higher than those associated with $f_{2}=1.98\;Hz$, $f_{3}=2.56\;Hz$, and $f_{5}=4.08\;Hz$, which collectively compose the spectral dynamic response of the structure (Figure 7, Top-left). Similarly, the lateral dynamic response at the 50.0% span location (midspan of the footbridge) also exhibited a dominant contribution from $f_{1}=1.07\;Hz$. However, the mode associated with $f_{3}=2.56\;Hz$ also contributed to the spectral response at this location, with an average participation of approximately 1.30% relative to the fundamental frequency (Figure 7, Top-right). In contrast, the vertical dynamic responses at both the 30.0% and 50.0% span location have a clear dominance of the second modal frequency $f_{2}=1.98\;Hz$. The means response amplitudes associated with this frequency exceeded 98.75% when compared with the lateral frequencies $f_{1}=1.07\;Hz$ and $f_{3}=2.56\;Hz$, which contributed marginally to the vertical dynamic response of the Premier Footbridge (Figure 7, Bottom, left and right). However, the overall magnitude of the structural response in the lateral direction was approximately 90.0% greater than that observed in the vertical direction, indicating that the vibration response of the structure was predominantly lateral.

The progressive increase in PDL during the gait tests produced variations in vibration amplitudes and slight shifts in the identified frequency values. These effects were particularly noticeable at the fundamental frequency $f_{1}=1.07\;Hz$, while the influence on higher modal frequencies was significantly smaller. This observation indicates that the fundamental mode was the modal component most affected in terms of both dynamic response amplitude and frequency variation as the TSs increased and their interaction with the structure intensified. Consequently, the HSI effects were mainly concentrated in the first mode of vibration. This behavior was observed even though the Premier Footbridge exhibits a dual dynamic sensitivity condition, with the first two modal frequencies ($f_{1}=1.07\;Hz$ and $f_{2}=1.98\;Hz$) located close to the fundamental spectral content of human walking.

**Figure 8:**
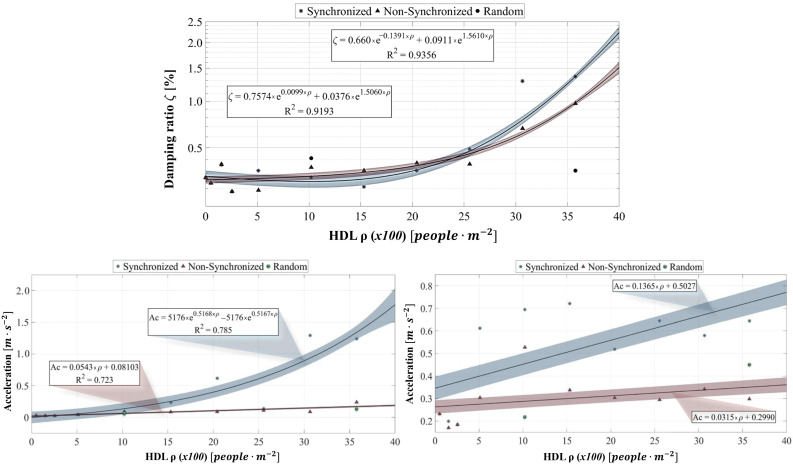
Premier Footbridge Performance due to pedestrian density loads (PDL). PDL ρ (×100) [$people\cdot m^{-2}$] vs. damping ratio ζ [%] (**Top**), PDL ρ (×100) [$people\cdot m^{-2}$] vs. lateral peak accelerations at the span center [$m\cdot s^{-2}$] (**Bottom-left**), and PDL ρ (×100) [$people\cdot m^{-2}$] vs. vertical peak accelerations at the span center [$m\cdot s^{-2}$] (**bottom-right**).

The concentration of HSI dynamic effects near the structural fundamental frequencies enabled the identification of pedestrian-induced damping on the Premier Footbridge, together with a decreasing tendency in the fundamental frequency $f_{n,1}$, mainly associated with lateral structural behavior. This reduction can be attributed to the additional effective mass introduced by the TSs during the tests, as shown in Figure 7. These HSI effects increased progressively with increasing PDL on the Premier Footbridge. For PDL lower than 0.256 $people\cdot m^{-2}$, the structural damping behavior of the first mode ($f_{n,1}$) was similar for both synchronized and non-synchronized gait tests. A comparable response was also observed for the random walking tests, in which participants were allowed to select their preferred direction and step frequency. However, beyond this PDL threshold, a clear divergence between test conditions was observed. In particular, the structural damping of the first mode increased significantly for the synchronized tests compared with the non-synchronized test using OMA assumptions as an approximately satisfied during all pedestrian excitations (stationary pedestrian occupancy). At the maximum evaluated PDL of 0.358 $people\cdot m^{-2}$, differences of 78.60% and 70.0% were identified for the synchronized and non-synchronized tests, respectively, considering prediction intervals (Figure 8, Top). It should be noted that synchronized pedestrian excitation does not fully satisfy the classical broadband white-noise assumptions commonly associated with OMA. Therefore, modal information estimates were interpreted as apparent operational modal properties under pedestrian-induced excitation conditions.

These damping values remain within the typical ranges expected for pedestrian bridges according to international vibration serviceability guidelines, such as SETRA [57] and HIVOSS [58]. Furthermore, the experimentally identified effective damping ratios values with PDL are comparable to the damping variations reported by HSI studies, around 1.0%, particularly those by Živanović et al. [59], Engolfsson et al. [49], and Riccardelli et al. [60]. On the other hand, the fitted trend curves presented in Figure 8 were obtained using nonlinear regression functions selected to represent the overall tendency of the experimental data. The associated prediction intervals correspond to 80.0% confidence bounds estimated from the regression residuals and were included to illustrate the dispersion and uncertainty associated with the measured responses. Due to the inherent variability of pedestrian-induced vibrations and human gait synchronization effects, the fitted relationships should be interpreted as indicative trends rather than deterministic predictive models.

The maximum vertical and lateral accelerations in the middle span of the footbridge (50.0% length) for the synchronized, non-synchronized, and random gait tests under different PDL values was determinate. For synchronized tests, lateral structure accelerations began to increase exponentially beyond a PDL of 0.10 people·m^−2^. In contrast, the non-synchronized and random walking tests exhibited an approximately linear trend. This difference resulted in acceleration values up to 85.42% higher for synchronized tests at the maximum evaluated density of 0.358 people·m^−2^ (Figure 8, Bottom-Left). Similarly, the vertical structural accelerations showed a nearly linear trend for all gait conditions. Nevertheless, higher acceleration levels were consistently observed for the synchronized tests compared with non-synchronized and random walking tests. At the maximum evaluated PDL of 0.358 people·m^−2^, the synchronized condition produced peak acceleration values up to 51.0% higher than those observed in the other test conditions (Figure 8, Bottom-Right). Also, both analyses were performed considering prediction intervals of 80.0%. Although a significant scatter is observed in the experimental measurements, consistent overall trends can still be identified for the analyzed response parameters. This variability is expected in pedestrian-induced vibration experiments due to the stochastic nature of human gait excitation, synchronization effects, and pedestrian–structure interaction mechanisms.

Overall, the experimentally observed structural behavior suggests that increasing pedestrian density levels may contribute to additional effective damping mechanisms while simultaneously increasing structural excitation levels, particularly under synchronized gait conditions, leading to larger vibration amplitudes and accelerations. The measured increase in effective damping and the concentration of HSI effects around the first lateral mode are consistent with previous investigations on dynamically sensitive pedestrian bridges subjected to synchronization phenomena [14,22,32,58]. However, unlike studies reporting significant interaction participation in multiple vibration directions, the present results indicate that the dual-frequency sensitivity of the Premier Footbridge does not necessarily lead to equivalent multiaxial HSI effects. Instead, the experimentally observed response remained predominantly governed by the first lateral mode, suggesting that the identified HSI behavior is strongly influenced by the specific structural characteristics of the investigated bridge, including its low apparent lateral modal mass, lightweight structural configuration, and the proximity between the first lateral natural frequency and typical pedestrian step frequencies.

### 3.2. Experimental Behavior of Human-Induced Gait Loads

**Figure 9:**
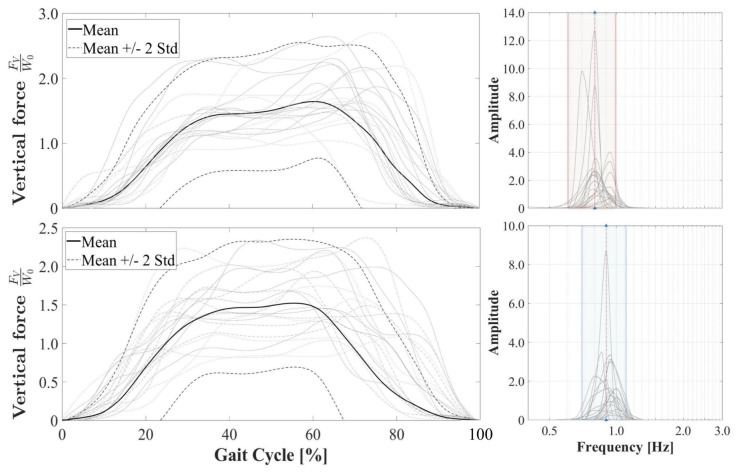
Vertical behavior of normalized human-induced gait loads ($F_{v}/W_{o})$ on rigid surface acquired by insole pressure sensor Stridalyzer Insight. $F_{v}/W_{o}$ with gait non-synchronized vs. gait cycle [%] (**Top-Left**), amplitude $F_{v}/W_{o}$—gait non-synchronized vs. frequency [Hz] (**Top-Right**), $F_{v}/W_{o}$ with gait synchronized vs. gait cycle [%] (**Bottom-Left**), amplitude $F_{v}/W_{o}$—gait synchronized vs. frequency [Hz] (**Bottom-Right**).

Human gait tests on rigid surfaces were conducted according to the procedures described in Section 2.2.4 and Section Rigid Surface Gait Test. The human-induced vertical gait loads, normalized with respect to the body weight of each TS ($F_{v}/W_{o}$), showed similar behavior for both synchronized and non-synchronized gait conditions. When comparing these two conditions, the mean values differed by less than 7.30%, with standard deviation bands lower than 27.30% (Figure 9, Top & Bottom-Left). In addition, the average gait frequencies for the synchronized (red) and non-synchronized (blue) tests differed by approximately 11.10%, with deviation bands below 20.0% (Figure 9, Top & Bottom-Right).

**Figure 10:**
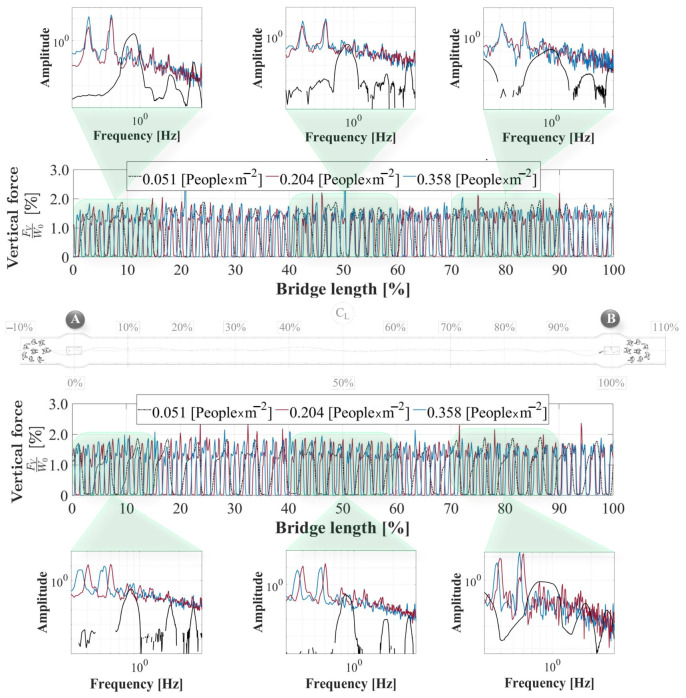
Behavior along the footbridge of the human-induced gait loads under three PDL (0.051, 0.204, and 0.358 $people\cdot m^{-2}$). Synchronized (**Top**) and non-synchronized (**Bottom**) gait tests.

Although a similar behavior of the human-induced vertical loads was observed on the Premier Footbridge for low PDL levels (approximately 0.05 people·m^−2^), comparable to those measured on rigid surfaces, noticeable changes appeared as the PDL increased. For PDL values greater than 0.15 people·m^−2^, variations in the amplitude of the human-induced loads and changes in pedestrian step frequency were identified during the dynamic gait tests for both gait conditions, as shown in Figure 10. These variations can be primarily attributed to human–human interaction (HHI) effects. Under synchronized gait conditions on the Premier Footbridge and for PDL values greater than 0.15 people·m^−2^, a dominant step frequency around 0.60 Hz was identified through spectral analysis of the experimental pedestrian loads acquired using insole devices. This represents a difference of close to 40.0% compared with the values measured on rigid surfaces. In contrast, under non-synchronized gait conditions, the dominant step frequencies varied with both the increase in PDL and the position along the structure, leading to differences exceeding 45.0% relative to rigid surface measurements.

**Figure 11:**
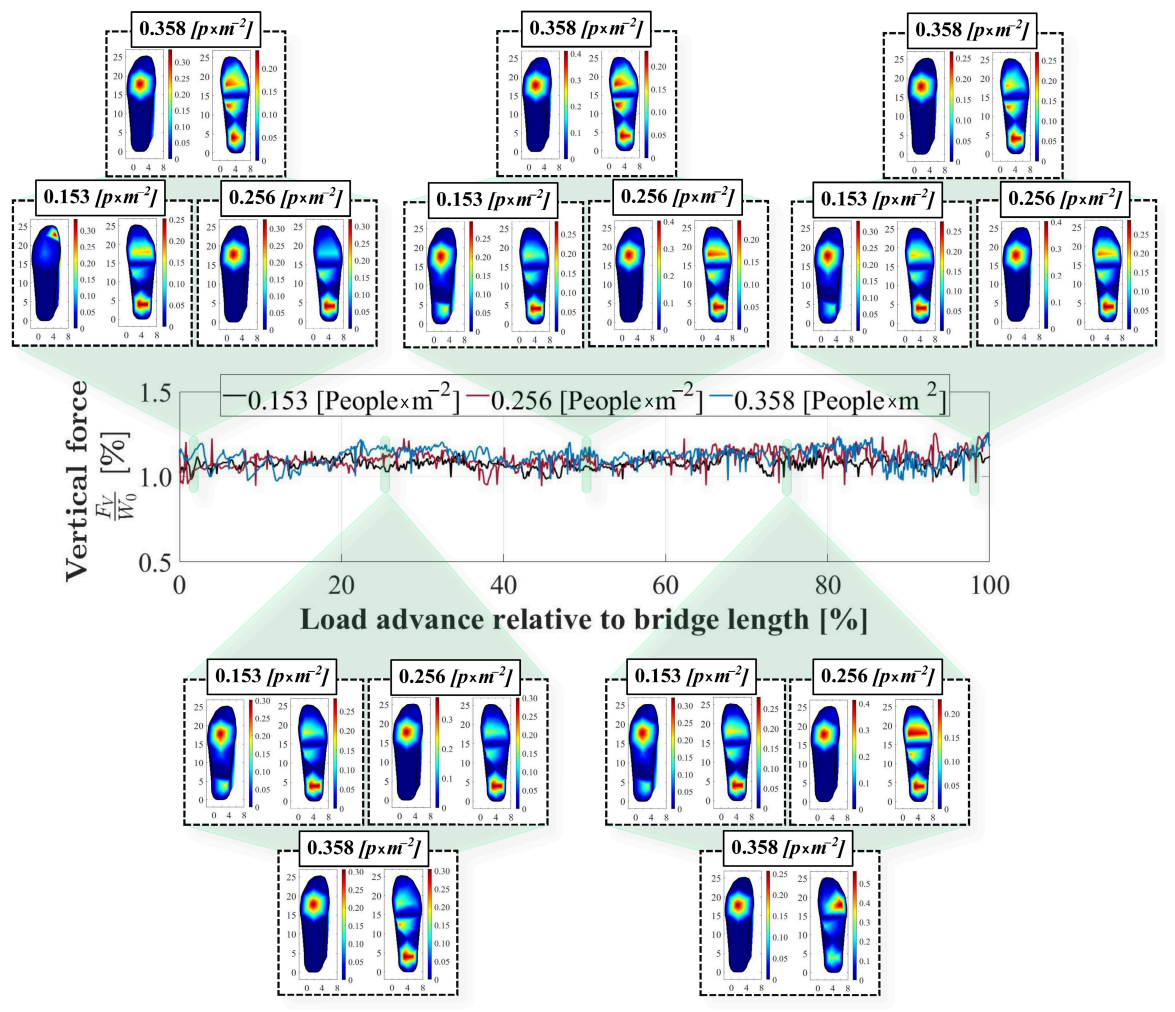
Performance of human-induced loads during a passive state (standing) in the middle span of the Premier Footbridge under three non-synchronized human gait tests with PDL of 0.153, 0.256, and 0.358 $people\cdot m^{-2}$.

Pedestrian-induced loads corresponding to passive state (standing pedestrians) were also identified at the midspan of the structure during the non-synchronized gait tests for PDL of 0.153, 0.256, and 0.358 people·m^−2^, as described in Section Gait Tests on Premier Footbridge. The vertical loads applied by these pedestrians remained approximately equal to their body weight, with variations lower than 10.0% for all evaluated PDL levels on the footbridge, as shown in Figure 11. However, internal Human–Structure Interaction (I-HSI) effects were identified. In these cases, pedestrians redistributed their internal body loads between both legs to maintain balance while the structure was vibrating dynamically, without producing significant changes in the total load transmitted to the bridge. The insole pressure sensors allowed the identification of this leg-by-leg load redistribution, revealing the balance adjustments adopted by pedestrians to maintain stability. Such behavior may generate dynamically sensitive conditions from the pedestrian perspective, potentially affecting the perceived serviceability of the structure. This study represents an initial step toward understanding I-HSI effects and their influence on structural serviceability.

The identified HSI trends confirm previous findings indicating that pedestrians may behave as active dynamic oscillators capable of modifying the dynamic properties of lightweight structures [15,22,58,59]. In particular, the observed increase in damping with increasing pedestrian density load (PDL) is consistent with prior analytical and experimental studies on pedestrian–structure interaction mechanisms. Nevertheless, the relatively limited interaction levels identified in the vertical direction differ from several conventional assumptions commonly adopted in simplified HSI models. These results suggest that the magnitude and distribution of interaction effects strongly depend on the modal properties and directional dynamic sensitivity of the investigated bridge.

### 3.3. Serviceability Performance of the Premier Footbridge

Over the past decades, structural vibrations generated by human-induced gait loads have been widely recognized as the main serviceability concern for pedestrian footbridges due to their recurrence and dynamic coincidence with human walking frequencies. This condition is largely associated with the high sensitivity of human perception to low levels of vibration during walking, which can be on the order of $1.0\cdot 10^{-3}\;m\cdot s^{-1}$ [61,62]. As a result, pedestrians act as the primary receivers of structural vibrations and therefore ultimately control the perceived serviceability of the structure. However, the intrinsic variability of human gait behavior, associated with factors such as body weight, step frequency, age, sex, activity level, and interaction with structural responses, influence the serviceability performance of footbridges. Reduced vibration serviceability may lead to discomfort, reduced usage of the structure, or, in some cases, the development of coupling phenomena with pedestrian loads such as Synchronous Lateral Excitation (SLE) [43,61,63,64,65]. The scientific interest of the community in understanding, quantifying, and modelling HSI effects in footbridges has led to the development of international standards that specify allowable acceleration limits to ensure adequate comfort levels for pedestrians. A brief review of allowable vertical and lateral comfort accelerations for different international standards is presented in Table 4.

**Figure 12:**
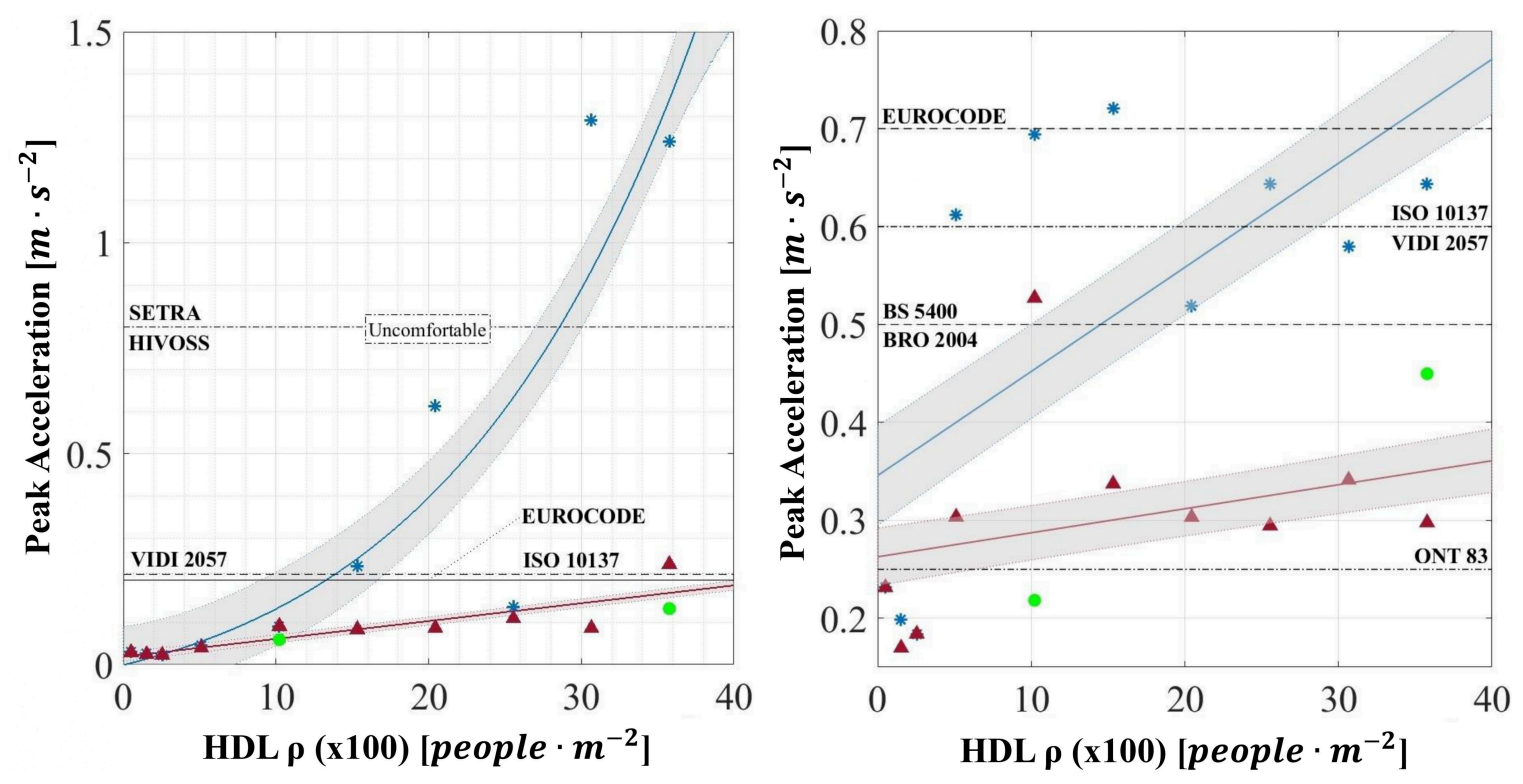
Premier Footbridge serviceability performance due to PDL compared to international standards/guidelines. PDL ρ (×100) [$people\cdot m^{-2}$] vs. lateral peak accelerations at middle span [$m\cdot s^{-2}$] (**Left**), and PDL ρ (×100) [$people\cdot m^{-2}$] vs. vertical peak accelerations at middle span [$m\cdot s^{-2}$] (**Right**). Synchronized gait tests (blue-mean), non-synchronized gait tests (red-mean), and random gait tests (green).

The comfort acceleration limits specified by these international standards/guidelines were evaluated for the Premier Footbridge at the midspan location. The modal frequencies used in the assessment ($f_{L}=1.06\;Hz$ and $f_{V}=1.98\;Hz$) were obtained from the OMA, as described in Section Operational Modal Analysis (OMA) and Modelling of the Structure. The lateral structural accelerations observed during all non-synchronized gait tests remained below the comfort limits defined in the evaluated standards. However, the lateral accelerations measured during synchronized gait tests exceeded the comfort limits proposed by SETRA and HIVOSS (around 0.80 $m\cdot s^{-2}$) for PDL values higher than 0.30 $people\cdot m^{-2}$, as shown in Figure 12 (Left). A similar trend was observed for vertical structural accelerations. For non-synchronized gait tests the measured accelerations remained below the comfort limits for all evaluated PDL values, with only slight exceedances relative to the strict ONT-83 criterion. In contrast, the vertical structural accelerations recorded during synchronized gait tests exceeded 0.50 $m\cdot s^{-2}$, which is considered uncomfortable according to most international standards, except for the Eurocode limit of 0.70 $m\cdot s^{-2}$ (Figure 12, Right).

The measured serviceability response under synchronized pedestrian loading agrees with previous studies demonstrating that crowd synchronization may substantially amplify lateral vibration levels in slender pedestrian bridges [28,30,32]. However, despite the multiaxial dynamic sensitivity of the structure, the most critical serviceability conditions remained primarily governed by lateral vibrations. This observation differs from investigations where vertical responses dominate pedestrian comfort assessments and suggests that the structural behavior of the Premier Footbridge is highly dependent on its specific modal configuration, modal separation, and susceptibility to lateral synchronization phenomena.

In addition, the multi-axial dynamic sensitivity of Premier Footbridge is particularly relevant, since the PDL values investigated in this study (up to 0.358 $people\cdot m^{-2}$) correspond to low-to-moderate PDL levels according to the SETRA and HIVOSS guidelines. At higher PDL commonly observed in operational conditions (around 0.80 $people\cdot m^{-2}$), stronger HSI effects may develop, potentially amplifying structural vibration responses and compromising the serviceability performance of the footbridge.

### 3.4. Premier Footbridge Benchmark Multiaxial Dataset

The Premier Footbridge Benchmark Multiaxial Dataset was developed to characterize the dynamic behavior of the structure under different PD conditions and gait patterns. The dataset includes synchronized measurements of multiaxial structural accelerations, pedestrian-induced loads, and environmental vibration conditions obtained during full-scale experimental tests. Such in situ experimental data are important for improving the understanding and validation of human–structure interaction (HSI) effects in dynamically sensitive footbridges [22,42,52,74].

The experimental results revealed measurable variations in modal properties and vibration response under different pedestrian occupancy conditions, particularly in the first lateral vibration mode. These characteristics make the dataset suitable for the evaluation of passive HSI effects and for the calibration of experimental or numerical HSI models based on measured structural responses [15,22,74,75,76]. In addition, the dataset includes synchronized, non-synchronized, and random walking conditions covering a wide range of PDL levels, which may also support future investigations on crowd-induced vibration mechanisms and pedestrian synchronization effects [30,60,77,78,79].

In brief, the dataset provides a full-scale experimental reference for future studies on pedestrian-induced vibrations, multiaxial structural response, and vibration serviceability assessment in lightweight pedestrian bridges. Additionally, the measured low-frequency multiaxial vibration responses obtained from the Premier Footbridge may also provide useful experimental references for future investigations involving nonlinear vibration systems and vibration-based energy harvesting technologies operating under pedestrian-induced excitation environments. In particular, the experimentally identified vibration characteristics under different pedestrian density and synchronization conditions may support the evaluation and validation of innovative low-frequency energy harvesting devices and nonlinear vibration mitigation systems subjected to human-induced dynamic excitation [80,81,82,83].

## 4. Conclusions

In this study, an extensive experimental assessment of human–structure interaction (HSI) effects was conducted on an operational urban footbridge with pronounced multiaxial dynamic sensitivity, the Premier Footbridge. The experimental tests were performed for pedestrian density loads (PDL) lower than 0.358 people·m^−2^, which correspond to low to moderate levels according to several international guidelines. Different gait conditions were evaluated, including synchronized, non-synchronized, and random gait patterns. The resulting Premier Footbridge dataset represents one of the first comprehensive experimental datasets aimed at evaluating serviceability performance under HSI effects in a multiaxially sensitive footbridge. The main conclusions of this study are summarized as follows:

1. The frequency-domain analysis of vertical and lateral structural accelerations measured at the 30.0% and 50.0% spans revealed that the dominant interaction effects were concentrated around the first lateral natural frequency (1.07 Hz). Although the Premier Footbridge exhibits multiaxial dynamic sensitivity due to the presence of both lateral (1.07 Hz) and vertical (1.98 Hz) fundamental frequencies within the characteristic excitation range of human walking, the experimental results demonstrated that the effective HSI response was predominantly governed by the first lateral mode. Therefore, in the context of this study, multiaxial dynamic sensitivity refers to the structural susceptibility to pedestrian-induced excitation in multiple vibration directions, rather than implying strong bidirectional dynamic coupling between lateral and vertical responses. As PDL increased, the structural response exhibited higher vibration amplitudes and a gradual reduction in the lateral natural frequency. Furthermore, the estimated cross-modal influence levels, calculated from the relative spectral participation of vertical and lateral responses measured under the evaluated gait conditions, remained below approximately 2.20%, indicating limited coupling between these vibration directions and suggesting that the observed HSI behavior is strongly influenced by the specific modal characteristics and dynamic configuration of the investigated bridge.
2. Structural damping ratios remained relatively stable for synchronized and non-synchronized gait conditions at PDL below 0.256 people·m^−2^. However, above this threshold, synchronized gait produced a significant increase in damping in the first vibration mode compared with non-synchronized conditions. At the maximum tested density of 0.358 people·m^−2^, the damping ratios increased by 78.6% for synchronized tests and 70.0% for non-synchronized tests. This increase in damping is associated with HSI effects, acting as an additional energy dissipation mechanism introduced by pedestrians. Nevertheless, the overall vibration levels of the system continued to increase with PDL, particularly under synchronized gait conditions, indicating that the growth in pedestrian-induced excitation exceeded the additional damping introduced by HSI effects.
3. Lateral accelerations exhibited a pronounced nonlinear increase under synchronized walking conditions once the load density exceeded 0.10 people·m^−2^, while non-synchronized and random walking tests showed a predominantly linear trend. This resulted in a difference of approximately 85.4% in the maximum lateral structural accelerations at a PDL of 0.358 people·m^−2^. Vertical accelerations, on the other hand, followed a nearly linear response for all gait conditions, although synchronized walking consistently produced higher amplitudes, leading to a 51.0% difference in maximum accelerations at the highest PDL. A comparison with international comfort criteria indicates that the lateral vibration behavior governs the serviceability performance of the structure, even under relatively low PDL.
4. The experimental dynamic gait tests revealed measurable variations in pedestrian gait behavior when PDL exceeded 0.15 people·m^−2^, suggesting the presence of HSI-related behavioral adaptations. Under synchronized walking conditions, a dominant step frequency emerged with variations of approximately 40.0% relative to reference gait conditions on rigid surfaces, while non-synchronized conditions exhibited variations exceeding 45.0%. Although these differences were primarily interpreted from an engineering and operational perspective rather than through formal statistical hypothesis testing, they indicate substantial modifications in pedestrian gait organization under pedestrian-induced vibration conditions. Despite these behavioral changes, the magnitude of pedestrian-induced loads in the passive state remained close to body weight, with variations below 10.0% across the evaluated densities. These observations suggest the presence of internal human–structure interaction (I-HSI) mechanisms, characterized by a redistribution of internal human loads without substantial changes in the externally applied structural loads.
5. The Premier Footbridge dataset provides highlights and a useful experimental base for improving current HSI models used to simulate pedestrian-induced vibrations. The dataset enables the investigation of both passive interaction effects, such as modifications in damping ratios and natural frequencies, and active interaction mechanisms associated with pedestrian gait adaptation to structural vibrations. Consequently, the data can contribute to the development and calibration of microscopic and macroscopic crowd-loading models, supporting future improvements in design guidelines and serviceability assessment procedures for pedestrian bridges.

Although the experimental campaign provides notable insight into HSI mechanisms, the results are limited to a single structural configuration and a controlled range of pedestrian densities. Future studies should investigate higher pedestrian densities and different structural typologies to generalize the observed interaction effects.

## Figures and Tables

**Table 1:** Experimental identified modal properties (mode number $\overset{˘}{ȷ}$, natural frequencies ${\hat{f}}_{j}$ and ${\overset{˘}{f}}_{j}$, and damping ratios $\hat{\xi }$ and $\overset{˘}{\xi }$) of all modes with a natural frequency below 20.0 Hz; and corresponding mode number j and natural frequencies $f_{m,j}$ predicted by the FE model, calculated MAC, and corresponding apparent modal mass in the vertical $m_{\Phi ,V}$ and lateral $m_{\Phi ,L}$ directions.

$\overset{˘}{ȷ}$ Mode	NExT-ERA		SSI				FE Model			
	$\hat{f}$ [Hz]	${\hat{f}}_{sd}$ [Hz]	$\overset{˘}{f}$ [Hz]	${\overset{˘}{f}}_{sd}$ [Hz]	$\overset{˘}{\xi }$ [%]	$\overset{˘}{\xi }$_sd_ [%]	$f_{m}$ [Hz]	MAC	$m_{\Phi ,V}$	$m_{\Phi ,L}$
1	1.07	0.02	1.06	0.01	0.77	0.41	1.07	0.99	Ƚ	114
2	1.98	0.06	1.95	0.05	0.86	0.52	1.96	0.99	114	℣
3	2.56	0.01	2.56	0.01	0.12	0.11	2.55	†*		
4	3.15	0.01	3.15	0.03	0.47	0.23	3.10	0.99	349	℣
5	4.08	0.01	4.09	0.03	0.18	0.03	4.14	0.98	Ƚ	155
6	6.65	0.02	6.67	0.01	0.48	0.09	6.65	0.92	Ƚ	137
7	7.81	0.03	7.81	0.03	0.65	0.13	7.76	0.73	250	℣
8	8.74	0.06	8.71	0.01	0.28	0.07				
9	12.15	0.10	12.12	0.02	0.47	0.12				
10	13.36	0.02	13.37	0.02	0.35	0.18				
11	15.50	0.19	15.47	0.05	0.94	0.21				
12	18.29	0.16	18.30	0.01	0.33	0.05				

†* → There was not sufficient experimental information to represent the modal form.

**Table 2:** Identification number (ID), height (H), body mass (BM), and sex (S, f→female or M→Male) of each test subject (TSs).

ID	H [m]	BM [kg]	S	ID	H [m]	BM [kg]	S	ID	H [m]	BM [kg]	S	ID	H [m]	BM [kg]	S
1	1.75	86.20	M	19	1.58	49.10	f	37	1.87	99.20	M	55	1.73	64.10	M
2	1.66	69.00	M	20	1.67	56.60	f	38	1.69	54.40	f	56	1.65	62.60	M
3	1.72	68.30	M	21	1.75	73.00	M	39	1.77	84.10	M	57	1.66	65.30	M
4	1.82	75.00	M	22	1.75	85.30	M	40	1.71	69.20	M	58	1.73	66.40	M
5	1.71	62.70	M	23	1.65	54.00	f	41	1.78	89.80	M	59	1.77	72.80	M
6	1.76	82.50	M	24	1.80	76.50	M	42	1.68	50.00	f	60	1.88	74.70	M
7	1.73	59.00	f	25	1.80	75.30	M	43	1.64	57.57	M	61	1.68	74.10	M
8	1.78	64.20	M	26	1.84	84.00	M	44	1.64	53.40	f	62	1.74	75.90	M
9	1.69	73.40	M	27	1.68	58.20	M	45	1.75	99.50	M	63	1.72	74.20	M
10	1.79	61.10	M	28	1.66	56.30	f	46	1.71	76.30	M	64	1.60	65.80	M
11	1.70	91.50	f	29	1.73	72.55	M	47	1.72	81.70	M	65	1.69	56.10	M
12	1.66	53.80	M	30	1.76	67.20	M	48	1.81	68.70	M	66	1.61	63.70	M
13	1.78	70.00	M	31	1.79	81.90	M	49	1.74	66.70	M	67	1.77	79.40	M
14	1.69	67.70	M	32	1.82	87.40	M	50	1.73	67.90	M	68	1.67	72.30	M
15	1.68	59.40	f	33	1.73	86.90	M	51	1.82	91.30	M	69	1.73	64.90	M
16	1.73	92.70	M	34	1.63	57.00	f	52	1.67	60.90	M	70	1.78	70.30	M
17	1.64	55.60	f	35	1.68	55.00	f	53	1.77	81.20	M				
18	1.74	67.50	M	36	1.66	53.60	f	54	1.71	60.50	f				

**Table 3:** Configuration of the Gait-test on Premier Footbridge.

Test No.	Gait Condition			Number of Test Subjects			Pedestrian Density Load (PDL) [$people\cdot m^{-2}$]
	Synchronized [$f_{g}\approx 2.0\;Hz$]	Non-Synchronized	Random	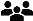 x n	℄ *	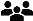 x m	
1	x			1	0	0	0.51%
2		x		1	0	0	
3	x			2	0	1	1.53%
4		x		2	0	1	
5	x			3	0	2	2.55%
6		x		3	0	2	
7	x			5	0	5	5.11%
8		x		5	0	5	
9	x			10	0	10	10.22%
10		x		10	0	10	
11			x	-	-	-	10.22%
12	x			15	0	15	15.33%
13		x		15	0	15	
14 *		x		15	1	15	15.33%
15	x			20	0	20	20.44%
16		x		20	0	20	
17	x			25	0	25	25.55%
18		x		25	0	25	
19 *		x		25	1	25	25.55%
20	x			30	0	30	30.66%
21		x		30	0	30	
22	x			35	0	35	35.77%
23		x		35	0	35	
24 *		x		35	1	35	35.77%
25			x	-	-	-	35.77%

* Pedestrian in a passive state (standing) at the middle span of the structure (50.0%).

**Table 4:** A brief review of international standard/guideline on comfort acceleration limits.

Standard/Guideline	Direction of Evaluation	
	Vertical [$m\cdot s^{-2}$]	Lateral [$m\cdot s^{-2}$]
BS 5400 [66]; BRO 2004 [67]	$0.5\sqrt{f_{v}}$	-
ONT 83 [68]	$0.25\sqrt{f_{v}}$	-
VIDI 2057 [69]	$0.6\sqrt{f_{v}}$	0.214
ISO 10137 [70]	$\left\{\begin{array}{c} 1.0\text{–}4.0\;\mathrm{Hz}\to \;\;\;\;0.70\text{–}0.1{0f}_{v}\;\;\;\;\\ 4.0\text{–}8.0\;\mathrm{Hz}\to \;\;\;\;\;0.70\\ 8.0\text{–}80.0\;\mathrm{Hz}\to \;\;\;\;\;0.0375f_{v} \end{array}\right.$	$\left\{\begin{array}{c} 1.0\text{–}2.0\;\mathrm{Hz}\to \;\;\;\;\;0.216\\ >2.0\;\mathrm{Hz}\;\to \;\;\;\;0.110f_{l} \end{array}\right.$
SETRA [57]; HIVOSS [58]	$\left\{\begin{array}{l} \mathrm{Minimum}\to \;\;\;\;\;1.0\text{–}2.50\\ \mathrm{Uncomfortable}\to \;\;\;>2.50 \end{array}\right.$	$\left\{\begin{array}{l} \mathrm{Minimum}\to \;\;\;\;\;0.30\text{–}0.80\\ \mathrm{Uncomfortable}\to \;\;\;>0.80 \end{array}\right.$
Eurocode 0 [71], 1 [72], and 5 [73].	$0.5\sqrt{f_{v}}\;\;\;or\;\;\;0.70$	$0.14\sqrt{f_{l}}\;\;\;or\;\;\;0.15$

## Data Availability

Raw data is available in https://doi.org/10.17632/4w3ytxrzxt.2. The rest of the processed data will be made available on request.

## Abbreviations

The following abbreviations are used in this manuscript:

HSI	Human–structure interaction
S2HI	Structure-to-human interaction
H2SI	Human-to-structure interaction
HHI	Human-to-human interaction
PDL	Realistic pedestrian density load
OMA	Operational modal analysis
FE	Finite element
PP	Peak-picking
MAC	Modal Assurance Criterion
TSs	test subjects
GPS	global positioning system
PSU	power supply unit
MOf	Microsoft Office
CPSD	Cross-Power Spectral Density
I-HSI	internal human–structure interaction
SLE	Synchronous Lateral Excitation
